# Postnatal growth restriction alters myocardial mitochondrial energetics in mice

**DOI:** 10.1113/EP091304

**Published:** 2024-01-05

**Authors:** Joseph R. Visker, Eric C. Leszczynski, Austin G. Wellette‐Hunsucker, Ashley C. McPeek, Melissa A. Quinn, Seong Hyun Kim, Jason N. Bazil, David P. Ferguson

**Affiliations:** ^1^ The Nora Eccles Harrison Cardiovascular Research and Training Institute University of Utah Salt Lake City Utah USA; ^2^ Department of Kinesiology Michigan State University East Lansing Michigan USA; ^3^ Department of Physiology University of Kentucky Lexington Kentucky USA; ^4^ Department of Physiology Michigan State University East Lansing Michigan USA

**Keywords:** cardiovascular disease, development, growth restriction, mitochondrial function, oxidative stress, reactive oxygen species

## Abstract

Postnatal growth restriction (PGR) can increase the risk of cardiovascular disease (CVD) potentially due to impairments in oxidative phosphorylation (OxPhos) within cardiomyocyte mitochondria. The purpose of this investigation was to determine if PGR impairs cardiac metabolism, specifically OxPhos. FVB (Friend Virus B‐type) mice were fed a normal‐protein (NP: 20% protein), or low‐protein (LP: 8% protein) isocaloric diet 2 weeks before mating. LP dams produce ∼20% less milk, and pups nursed by LP dams experience reduced growth into adulthood as compared to pups nursed by NP dams. At birth (PN1), pups born to dams fed the NP diet were transferred to LP dams (PGR group) or a different NP dam (control group: CON). At weaning (PN21), all mice were fed the NP diet. At PN22 and PN80, mitochondria were isolated for respirometry (oxygen consumption rate, $J_{O_{2}}$) and fluorimetry (reactive oxygen species emission, $J_{H_{2}O_{2}}$) analysis measured as baseline respiration (LEAK) and with saturating ADP (OxPhos). Western blotting at PN22 and PN80 determined protein abundance of uncoupling protein 3, peroxiredoxin‐6, voltage‐dependent anion channel and adenine nucleotide translocator 1 to provide further insight into mitochondrial function. ANOVAs with the main effects of diet, sex and age with α‐level of 0.05 was set a priori. Overall, PGR (7.8 ± 1.1) had significant (*P* = 0.01) reductions in respiratory control in complex I when compared to CON (8.9 ± 1.0). In general, our results show that PGR led to higher electron leakage in the form of free radical production and reactive oxygen species emission. No significant diet effects were found in protein abundance. The observed reduced respiratory control and increased ROS emission in PGR mice may increase risk for CVD in mice.

## Abstract (highlights)

- **What is the central question of this study?**
  Postnatal growth restriction (PGR) in early life is associated with cardiovascular disease: what are the mechanisms with regard to oxidative phosphorylation (OxPhos)?
- **What is the main finding and its importance?**
  PGR during development reduces cardiac metabolism through impairments in OxPhos. Our results show the PGR heart has higher electron leakage in the form of free radical production and reactive oxygen species emission. These findings are associated with an increased risk for cardiovascular disease in adulthood. Simply put, a brief period of growth restriction in early life has long‐lasting effects on cardiac metabolism in adulthood.

## 1 INTRODUCTION

The Developmental Origins of Health and Disease (DOHaD) hypothesis suggests that growth‐restricted children have a higher risk for chronic disease in adulthood (Barker, 1997). Specifically, 7 million cardiovascular (CV)‐related deaths in adulthood occur each year due to growth restriction in early life (Baum et al., 2003). The COVID‐19 pandemic has reduced food availability, and worsened dietary practices, which likely increases the prevalence of postnatal growth restriction (PGR) (Elbehery et al., 2020; Jafri et al., 2021). Therefore, the health outcomes of growth restriction are relevant to the current CV health crisis facing the world today. Previous mouse research that modelled growth restriction using a diet manipulation strategy revealed that mice subjected to PGR (restriction on day of birth to weaning: PN1–21) have reduced left ventricular (LV) mass, reduced stroke volume and diastolic dysfunction in adulthood (PN70) (Ferguson et al., 2019; Pendergrast et al., 2020). Additionally, cardiomyocytes from PGR mice have impaired calcium flux (Ferguson et al., 2019) resulting in cardiac dysrhythmias in adulthood, all of which may stem from impaired cardiac metabolism and reduced oxidative phosphorylation (OxPhos) (Visker et al., 2018). Impairments to mitochondrial function, such as increased reactive oxygen species (ROS) emission and reduced re‐synthesis of ATP alter OxPhos and contribute to cardiovascular disease (CVD) (Badolia et al., 2020). Previous evidence has demonstrated that PGR mouse hearts at PN21 have reduced protein abundance of peroxiredoxin‐6 (PRDX6), which mitigates ROS emission through anti‐oxidative properties (Ma et al., 2016; Visker et al., 2020). Yet, there is negligible research exploring OxPhos of growth‐restricted hearts. Moreover, there is minimal research exploring the postnatal environment, which leaves a chasm in the DOHaD literature since PGR is strongly associated with myocardial impairments (Ferguson et al., 2019; Pendergrast et al., 2020; Visker et al., 2018).

For the heart to contract, cardiomyocytes require a constant supply of energy metabolites provided by mitochondria in the form of ATP (Sheeran & Pepe, 2017). The majority of ATP (>95%) utilized in the heart is synthesized via OxPhos, and poor mitochondrial function is a leading driver of cardiac pathology (Balaban, 2009). Substrate utilization in the fetal mammalian heart relies primarily upon carbohydrate‐sourced respiratory fuels (Lopaschuk et al., 1991). A metabolic transition occurs soon after birth, and the mature non‐growth‐restricted myocardium obtains ∼60–90% of its ATP from fatty acid oxidation, with the remaining balance stemming from carbohydrate metabolism (∼10–30%) (Taegtmeyer et al., 2005). The myocardial energetics literature reports that poor mitochondrial function is a leading driver of cardiac pathology (Lopaschuk et al., 1993) and is often accompanied by a reduction in metabolic flexibility and an increase in oxidative stress. Thus, these metabolic factors may contribute to the PGR‐mediated effects of CVD later in life.

The purpose of the present investigation was to determine the effects of PGR on myocardial mitochondrial function in vitro. Since the healthy myocardium is considered a ‘metabolic omnivore’, we used three major catabolic pathways to assess PGR effects on mitochondrial metabolism: (1) pyruvate and malate (complex I: P/M), (2) succinate and rotenone (complex II: S/R), and (3) palmitoylcarnitine and malate (fatty acid oxidation: PC/M). We tested whether PGR would alter OxPhos during early and adult murine life. Additionally, we examined if ROS emission was added to the pathophysiology of CVD seen in adulthood. Furthermore, we hypothesized that PGR would disrupt key proteins involved in OxPhos such as uncoupling protein 3 (UCP3), PRDX6, voltage‐dependent anion channel (VDAC) and adenine nucleotide translocator 1 (ANT1). We found that PGR contributes to altered myocardial mitochondrial energetics during development (PN22) and again in adulthood (PN80).

## 2 METHODS

### 2.1 Ethical approval

All experiments were completed in accordance with the *Guide for the Care and Use of Laboratory Animals* and were approved by the Institutional Animal Care and Use Committee at Michigan State University (PROTO202100230). Additionally, we followed the principles and regulations for animal experiments outlined by Grundy (2015). All animals were housed in a vivarium on wood‐chip bedding in a 21°C room with a 12 h light–dark cycle.

### 2.2 Nutritive model

**FIGURE 1:**
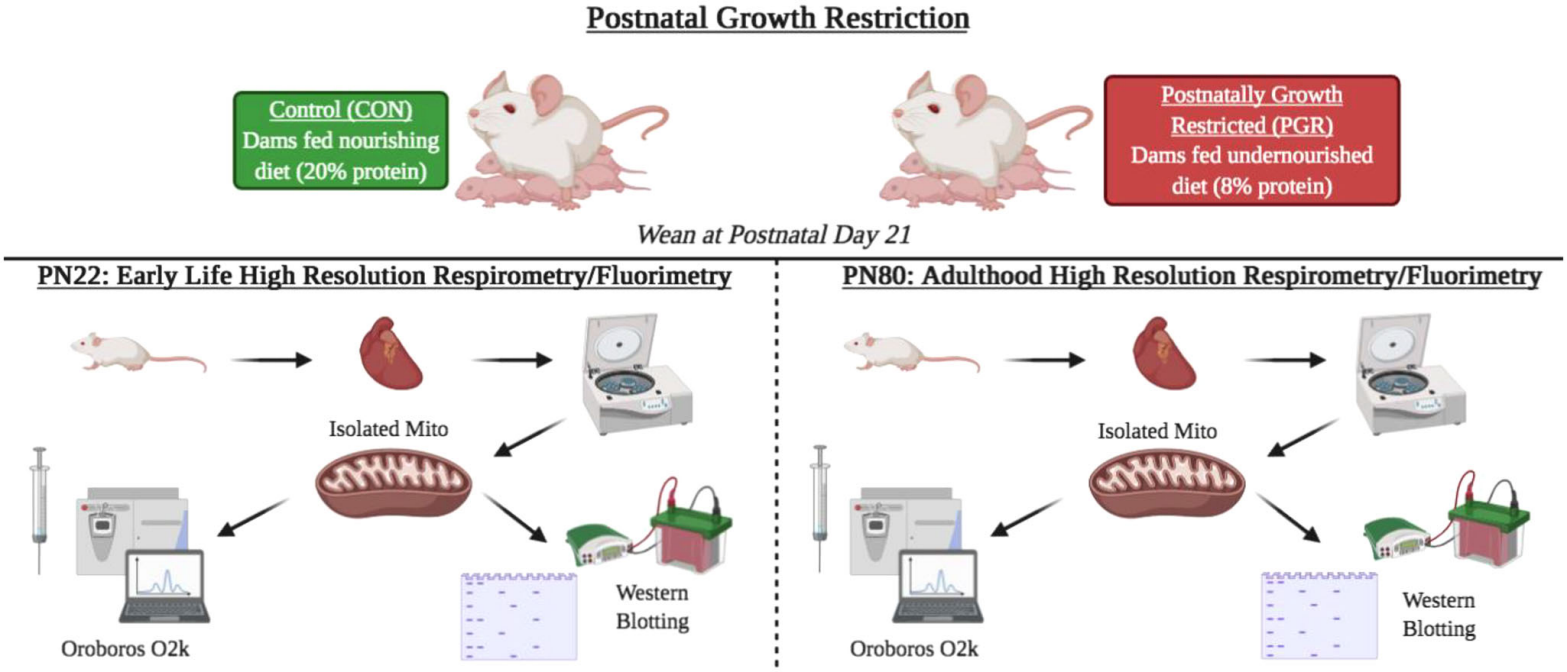
Experimental design. Two weeks before breeding, dams were given access to a normal protein diet (NP: 20% protein) or isocaloric low protein diet (8% protein). CON born pups were pooled and cross‐fostered to a different NP‐fed dam (CON group; *n* = 10 litters) or a different low protein fed dam (PGR group; *n* = 10 litters); the pups born to LP dams were not used in this study. At PN21, all mice were weaned to the NP diet, and thus postnatal growth restriction was isolated to the developmental window of lactation. Following a day of rest, at PN22 (1 day post‐weaning), cohorts of mice were killed, and hearts were surgically removed. After this, mitochondria were isolated for respirometry ($J_{O_{2}}$) and fluorimetry ($J_{H_{2}O_{2}}$) flux measurements. This process was repeated with the remaining littermates at PN80. Additionally, western blotting was completed at both PN22 and PN80 to determine the relative protein abundance of essential mitochondrial proteins during development and adulthood.

Following the guidelines for DOHaD mouse study design (Dickinson et al., 2016), PGR was reproducibly induced in FVB (Friend Virus B‐type) mice using diet manipulation (Figure 1) (Ferguson et al., 2019; Fiorotto et al., 2014; Leszczynski et al., 2019; Pendergrast et al., 2020; Visker et al., 2018). Third‐parity FVB dams (Charles River Laboratories, Wilmington, MA, USA) consumed a semi‐purified normal protein (NP) diet (D06041301: 20% protein; Research Diets, New Brunswick, NJ, USA) based on AIN93G, or a low‐protein (LP) isocaloric diet (D06041302: 8% protein; Research Diets) 2 weeks prior to breeding (the composition of the research diets has been explained previously) (Visker et al., 2018). Dams that consume a LP diet yield 15–20% less milk with lower free amino acid and higher fatty acid content than controls, causing the suckling pups to have an 18% reduction in body mass (Crnic & Chase, 1978; Grigor et al., 1987). Breeding was achieved by placing one FVB male in a female cage for a 24‐h period, so all pups born were of comparable age. On the day of birth (PN1), pups born to dams fed the control diet were pooled together, weighed, sexed and cross‐fostered for equal distribution to two experimental groups: (1) control (CON) group: pups born to a control dam then cross fostered to a different control dam (*n* = 10 litters); and (2) postnatal growth restriction (PGR) group: pups born to a control dam then cross fostered to a dam fed the LP diet (*n* = 10 litters). The cross‐fostering method for this project resulted in a high rate of success with no rejection of the pups by the dams. Pups born from LP dams were not used in this study since they are gestationally growth restricted and not the focus of the present investigation.

Each litter consisted of eight pups (four males, four females) and individual pups within a litter were given tattoo identification. All litters were made equal in size, sex ratio and starting body mass. If needed, litter size was conserved throughout lactation by using ‘donor’ pups of similar age and weight to supplant any fatalities; these pups were excluded from this study. On PN21, all mice were weaned to the NP diet. At PN22, a sub‐cohort of a total of 16 mice (PN22–CON–Male (*n* = 4), PN22–CON–Female (*n* = 4), PN22–PGR–Male (*n* = 3) and PN22–PGR–Female (*n* = 5)) was placed under 1% isoflurane anaesthesia and killed by cervical dislocation, and then myocardial mitochondrial samples were analysed by high‐resolution respirometry and fluorimetry (see below). The remaining littermates remained on the NP diet from PN22 until PN80. Thus, PGR was isolated to the developmental window of lactation (PN1–PN22). At PN80, another sub‐cohort of 16 mice (PN80–CON–Male (*n* = 3), PN80–CON–Female (*n* = 6), PN80–PGR–Male (*n* = 4) and PN80–PGR–Female (*n* = 3)) were killed using the same technique described above for PN22. Hearts were again removed, weighed and used for respirometry and fluorimetry using isolated myocardial mitochondria. Heart mass is represented as absolute and standardized to body surface area (BSA), using Meeh's formula (Gouma et al., 2012):

$$\mathrm{Body}\;\mathrm{surface}\;\mathrm{area}=9.662\times {\left(\mathrm{body}\;\mathrm{weight}\left(g\right)\right)}^{0.667}$$

Body surface area standardization is a method commonly used to compare mice of different body masses (Gouma et al., 2012). Digital Vernier calipers (General Tools, Secaucus, NJ, USA) determined tibia length as an indicator for lean tissue growth (Lang et al., 2005). In total, 32 mice were used for respirometry and fluorimetry experiments across both time points of PN22 (*n* = 16 mice) and PN80 (*n* = 16 mice).

### 2.3 Isolation of myocardial mitochondria and protein quantification

Myocardial mitochondria were isolated from FVB mouse hearts at two developmental ages (PN22: 1 day after weaning; and PN80: adulthood) using differential centrifugation. All spins were completed at 4°C for 10 min as published previously (Bazil, 2017). Mice were placed under 4% isoflurane anaesthesia, until unresponsive to a physical stimulus. Following killing by cervical dislocation, the heart was removed and rinsed in cold cardioplegic solution (25 mM KCl, 100 mM NaCl, 10 mM dextrose, 25 mM MOPS and 1 mM EGTA, 0.22 μm sterile filtered at pH 7.5). Hearts were then washed and minced in cooled isolation buffer (200 mM mannitol, 50 mM sucrose, 5 mM dibasic potassium phosphate, 5 mM MOPS, 1 mM EGTA and 0.1% BSA at pH 7.15). After mincing with scissors, the nearly 1 mm^3^‐sized tissue sections were placed in a 50 mL canonical tube containing 25 mL of isolation buffer and 0.5 U/mL of protease (*Bacillus licheniformis*). The solution was homogenized at 18,000 rpm for 20 s with a 110 mm‐probe Omni homogenizer (Omni Inc., Kennesaw, GA, USA). Following homogenization, the first spin occurred at a speed of 8000 *g* in a table‐top centrifuge resulting in a pellet. The supernatant was discarded, and the pellet was rinsed and re‐suspended by vortexing in isolation buffer (25 mL), then centrifuged for a second time at a slower speed of 800 *g*. Following this spin, the supernatant was placed into a clean 50 mL Falcon tube, and the pellet was discarded. A third and final spin of 8000 *g* produced a mitochondrial‐rich pellet that was always kept on ice and was never used for more than 8 h. The pellet was then reconstituted in a small volume of isolation buffer (10–20 μL). The bicinchoninic acid (BCA) assay (Walker, 1994) with bovine serum albumin standards was used for determining total mitochondrial content using a Synergy H1MG plate reader (Biotek, Winooski, VT, USA).

Based upon the total concentration, all stock samples were then standardized to a working concentration of 30 mg/mL using isolation buffer. This allowed for comparisons across all experimental groups and time points, since the working concentration of samples was equal. To quantify cardiac mitochondrial content, we used the concentration of the mitochondrial pellet (mg/mL), heart mass (grams) and resuspension volume (30–40 μL). Briefly, the mitochondrial content was calculated by dividing the concentration of the mitochondrial yield (undiluted concentration multiplied by resuspension volume) by the weight of the heart, thus providing an estimation of the amount of mitochondrial protein per unit of heart tissue (Garnier et al., 2003).

### 2.4 High‐resolution respirometry and fluorimetry

Oxygen consumption rates ($J_{O_{2}}$, nmol mg^−1^ min^−1^) were measured at 37°C using an Oroboros Oxygraph 2K high‐resolution respirometer system (Oroboros Instruments, Innsbruck, Austria). The oxygraph uses an amperometric sensor to determine O_2_ consumption and concentration in a closed chamber. CON and PGR myocardial mitochondria were injected into the oxygraph machine with oxygenated respiration buffer (5 mM ATP, 5 mM dibasic potassium phosphate, 6 mM magnesium chloride, 130 mM potassium chloride, 1 mM EGTA, 20 mM MOPS, and 1% w/v BSA at pH 7.1). Experimental conditions varied only with the presence of 5 mM sodium pyruvate and 1 mM l‐malate (P/M), 5 mM succinate and 1 μM rotenone (S/R), or 25 μM palmitoyl‐carnitine and 1 mM l‐malate (PC/M). Malate was added to pyruvate and palmitoylcarnitine to avoid depletion of oxaloacetate. EGTA (1 mM), a calcium‐chelating agent, was added to avoid calcium effects (Bazil et al., 2010).

**FIGURE 2:**
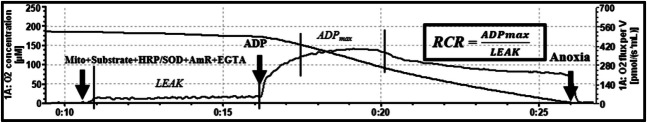
Representative oxygraph recording for $J_{O_{2}}$. Various substrates were loaded into the oxygraph chambers (P/M, S/R or PC/M). Next, horse radish peroxidase (HRP) and superoxide dismutase (SOD) were added since the Amplex Ultrared (AmR) to H_2_O_2_ reaction is catalysed by HRP and the main species of mitochondrial ROS produced is superoxide anion which is rapidly converted to H_2_O_2_ by SOD. Then, AmR is added as a fluorescent probe for H_2_O_2_ detection via the O2k‐Fluorometer. Next, EGTA was added as a calcium‐chelating agent. Cardiac mitochondria (CON or PGR; 30 mg/mL) were injected into the chambers for the measurement of LEAK state respirometry (5 min). A bolus of ADP was then added to stimulate mitochondrial respiration until the chamber becomes anoxic. The measurement of respiratory control ratio (RCR; unitless ratio) is computed by dividing the maximally stimulated ADP respiration (ADP_max_) by the LEAK state. The left *y*‐axis is the concentration of oxygen in the chamber (μM), while the right *y*‐axis is the $J_{O_{2}}$ flux (pmol/(s mL)). The unit of pmol/(s mL) is converted into nmol/mg/min. The *x*‐axis is time, as measured in minutes.

Our experiments involving mitochondrial substrate utilization (P/M, S/R and PC/M) were performed via respirometry (Figure 2). We define the following terms, as has previously been described (Vinnakota et al., 2016): (1) LEAK state where mitochondria respire to maintain resting membrane potential in the absence of ADP (Pesta & Gnaiger, 2012) and (2) ADP‐stimulated maximal respiration by the addition of 0.5 mM ADP (Vinnakota et al., 2016). From this, the respiratory control ratio (RCR) was calculated (LEAK/ADP respiration). The classic method used to calculate respiratory control (state 3/state 4) is not advised as state 4 is a respiratory condition that is determined by the amount of exogenous ATPases that co‐purify with mitochondria, which differs from lab to lab (Bazil et al., 2016, 2010; Doerrier et al., 2018; Duong et al., 2021; Vinnakota et al., 2016).

PGR mouse hearts are reported to have reduced levels of antioxidant enzymes, mainly PRDX6, which may indicate impairments to cytosolic and mitochondrial ROS handling (Visker et al., 2020). Thus, ROS emission ($J_{H_{2}O_{2}}$) was measured through fluorometry using the well‐establish horseradish peroxidase (HRP) hydrogen peroxide assay (AmpUR; Amplex Ultrared, Thermo Fisher Scientific, Waltham, MA, USA) (Duong et al., 2020). AmpUR was dissolved to a stock concentration of 10 mM according to the manufacturer's guidelines. Hydrogen peroxide calibration curves were made by the addition of 500 nM boluses using a stock solution of 200 μM, which were freshly prepared on the day of experiments. Superoxide dismutase and Type II HRP were separately dissolved to the stock concentration of 500 U/mL. $J_{H_{2}O_{2}}$ detection was performed using 10 μM AmpUR, 1 U/mL HRP and 5 U/mL superoxide dismutase in the chambers. Only stable portions of the amperometric $J_{H_{2}O_{2}}$ curves were measured, and any noticeable artifact in the recording was excluded.

### 2.5 Western blotting

We probed for mitochondrial proteins associated with LEAK‐$J_{O_{2}}$ and ROS to test the hypothesis that altered protein abundance influenced respiratory control. For western blotting we used a separate cohort of mice than used for fluorimetry and respirometry. Hearts from littermates at PN22 and 80 were homogenized in Norris buffer (HEPES, β‐glycerophosphate, ATP, protease inhibitor cocktail, benzamidine, phenylmethylsulfonyl fluoride, EDTA, MgCl_2_), as previously published (Shimkus et al., 2018). Next, samples were centrifuged to isolate the homogenate, and protein concentration was estimated through a BCA assay (Thermo Fisher Scientific) to allow for equal protein loading. Samples were loaded on a 10% SDS polyacrylamide gel and separated by electrophoresis. Proteins were then transferred to a Millipore (Burlington, MA, USA) Immobilion‐FL polyvinylidene difluoride membrane and allowed to air dry. Following the drying, membranes were re‐activated with methanol and dyed with LI‐COR (Lincoln, NE, USA) total protein stain to control for total protein abundance loaded on the gel. Membranes were then de‐stained, rinsed in Tris‐buffered saline solution with tween (TBS‐T), and blocked in 5% non‐fat dried milk for 1 h. Membranes were rinsed again in TBS‐T, and then incubated overnight with the primary antibodies for ANT1 (cat. no. ab110322, Abcam, Waltham, MA, USA), UCP3 (cat. no. ab180643, Abcam), VDAC (cat. no. 4866, Cell Signaling Technology, Danvers, MA, USA) and PRDX6 (cat. no. ab73350, Abcam) per the manufacturers’ specified instructions.

The following day, PRDX6, VDAC and UCP3 membranes were rinsed in TBS‐T, then incubated for 1 h with LI‐COR infrared secondary antibody (IRDye 800CW goat anti‐rabbit). Membranes were rinsed in TBS‐T and imaged on a LI‐COR Odyssey Fx Imager for infrared fluorescence. Following the manufacturer's recommendations, PN22 ANT1 membranes were given a 2‐day primary antibody incubation, rinsed in TBS‐T, then the same procedures described above were followed.

ANT1 membranes were also rinsed in TBS‐T the morning after primary antibody incubation, followed by an incubation with a chemiluminescent secondary antibody (cat. no. Ab205718, Abcam). Membranes were then rinsed in TBST, followed by a brief incubation in SignalFire ECL reagent (Cell Signaling Technology). ANT1 membranes were immediately imaged utilizing a LI‐COR Odyssey Fx Imager measuring chemiluminescence.

All images were exported to Empiria software (LI‐COR) to quantify band intensity by comparing target protein intensity to intensity from total protein stains, and results are expressed as arbitrary units (AU). Total protein staining for the normalization of western blotting is recognized for its reliability and reproducibility, providing a consistent method to account for variability in protein loading and transfer across samples. All quantitative analyses were conducted using the original raw files produced by the LI‐COR software, which analyses band intensity independently of the background, allowing for accurate comparisons between samples (Aldridge et al., 2008; Collins et al., 2015; Eaton et al., 2013; Ghosh et al., 2014; Maloy et al., 2022).

### 2.6 Statistics

At least three biological replicates were performed for each experimental condition. The Shapiro–Wilk and D'Agostino–Pearson omnibus tests were used to verify the normality of data. Grubbs's test (Prism v7.01, GraphPad Software, San Diego, CA, USA) was used to identify any significant outliers, and if identified they were removed prior to analysis. If data were not normally distributed, a logarithmic transformation was performed, following which a linear model and repeated measures ANOVA were used to compare growth curves between the CON and PGR groups (GraphPad Prism, v7.01). All values are expressed as means ± SD. Two‐way ANOVAs were used with the main effects of diet (CON vs. PGR) and sex (male vs. female) to compare heart mass (absolute or standardized) and tibia length. Three‐way ANOVAs were used for $J_{O_{2}}$ and $J_{H_{2}O_{2}}$ with the main effects of diet (CON vs. PGR), sex (male vs. female) and age (PN22 vs. PN80). Two‐way ANOVAs were used for western blot data with the main effects of diet (CON vs. PGR) and sex (male vs. female). To estimate our sample sizes needed we used pilot data respirometry and fluorimetry and conducted a power analysis achieve 80% power (1 − β). An α‐level of *P* < 0.05 was set a priori, and if necessary, a Tukey's HSD *post hoc* test was used for multiple comparisons.

## 3 RESULTS

### 3.1 Body mass

#### 3.1.1 PN1–22

**FIGURE 3:**
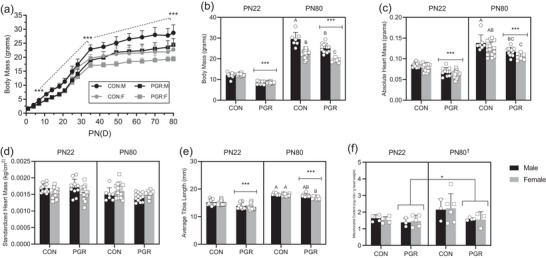
Influence of postnatal growth restriction on body mass, heart mass, tibia length and mitochondrial content. (a) Growth curve from PN1 to P80. Starting at PN7 through PN22, growth restriction significantly (*P* < 0.001) lowered the body mass of the PGR when compared with the CON group, with no sex effect. At PN25, PGR continued to cause significant (*P* < 0.001) reductions in body mass until PN80. Sex differences started at PN28 and continued until PN80 (*P* < 0.001), with males being larger than females. Repeated measures ANOVA was used. (b) Final body mass at respirometry (PN22 and PN80). Final body mass on the days of PN22 respirometry was significantly reduced (*P* < 0.001) in the PGR when compared with the CON group, with no sex effect. Final body mass on the day of PN80 respirometry was also significantly reduced (*P* < 0.001) in the PGR when compared with the CON group. A sex effect was present at PN80 with males being larger than females (*P* < 0.001). (c) Absolute heart mass (PN22 and PN80). The PN22–PGR hearts were significantly less (*P* < 0.001) than the CON group, with a sex effect present (males larger than females; *P* = 0.007). The PN80–PGR hearts were significantly reduced (*P* < 0.001) when compared to CON hearts, with a similar significant sex effect (*P* < 0.007). (d) Standardized heart mass (PN22 and PN80). There is no significant difference at PN22 or PN80, and there was no sex effect present. (e) Tibia length (PN22 and PN80). PGR significantly reduced (*P* < 0.001) average tibia length at PN22 when compared to CON group, with no sex effect present. At PN80, tibias of PGR were significantly less (*P* < 0.001) than the CON group, but only PGR–F (*P* < 0.001) were significantly reduced from controls through *post hoc* analysis. (f) Mitochondrial content (PN22 and PN80). PGR significantly reduced (*P* = 0.04) mitochondrial content standardized to heart weight when compared to CON. Additionally, there was an age effect (*P* = 0.04) with higher mitochondrial content present at PN80 when compared to PN22. Values are means ± SD. Two‐way and three‐way ANOVAs were used with the main effects of age (PN22 or PN80), diet (CON vs. PGR) and sex (male vs. female) with a Tukey HSD multiple comparison test for multiple comparisons. Levels within the figure not connected by the same letter (A, B or C) represent statistical significance (*P* < 0.05). ***Significantly different from CON group (*P* < 0.001). †Effect of age, representing that PN80 is significantly different from PN22.

Starting at PN7 body mass was significantly (*P* < 0.001) reduced in the PGR mice compared to CON mice. This result continued through weaning at PN21 (Figure 3a). On days of respirometry and fluorimetry (PN22), body mass remained significantly reduced (*P* < 0.001) in the PGR mice compared to the CON mice. There were no sex differences seen during the PN1–22 early window of development (Figure 3b).

#### 3.1.2 PN22–80

As seen in Figure 3a, PGR significantly (*P* < 0.001) reduced body mass at every weighing throughout the experiment from PN22 to PN80. At PN28 a significant sex effect emerged showing males weighed more than females (*P* < 0.001). Final body mass (Figure 3b; PN80) of the PGR mice was significantly reduced (*P* < 0.001) compared to controls.

### 3.2 Heart mass, tibia length and mitochondrial content

At PN22, absolute heart mass (Figure 3c) of PGR hearts was significantly reduced (*P* < 0.001) when compared to CON at PN22. At PN80, absolute heart mass of PGR mouse hearts was also significantly reduced (*P* < 0.001) when compared to CON hearts. At PN80, but not PN22, there was a sex effect with male hearts weighing more than female (*P* = 0.007). When heart mass was standardized at PN80, there were no significant differences between groups.

When heart mass was standardized to body surface area at PN22 and PN80, there was no more diet effect (Figure 3d), indicating that PGR hearts were proportional to the size of the mouse when compared to CON.

At PN22, the PGR mice had significantly reduced (*P* < 0.001) tibia lengths (Figure 3e) when compared to the CON mice. There was no sex effect at PN22 for tibia length (*P* = 0.080). At PN80, tibia length remained significantly reduced (*P* < 0.001) in the PGR mice. *Post hoc* analysis revealed that tibia length was only significantly reduced in the PGR–F mice compared to controls (both male and female comparisons; *P* < 0.001). Additionally, there was a significant sex effect in adulthood, with males having a longer tibia length than females (*P* < 0.001).

Across both timepoints combined (PN22 and PN80), the mitochondrial content of PGR mice was significantly reduced (*P* = 0.043, Figure 3f) when compared to the CON mice. Additionally, there was a significant effect of age (*P* = 0.047) with more mitochondrial content present at PN80 when compared to PN22.

### 3.3 Respirometry ($J_{O_{2}}$) and fluorimetry ($J_{H_{2}O_{2}}$)

#### 3.3.1 Pyruvate/malate

**FIGURE 4:**
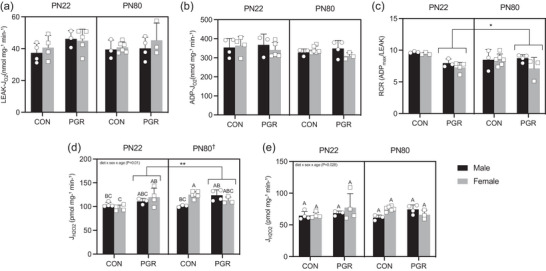
Respirometry ($J_{O_{2}}$) and fluorimetry ($J_{H_{2}O_{2}}$) with pyruvate/malate (P/M: NADH‐linked, complex I) in CON and PGR at PN22 and PN80. (a) LEAK‐$J_{O_{2}}$ in the PGR group was elevated (*P* = 0.068), but non‐significant when compared to CON. (b) During ADP‐$J_{O_{2}}$, there were no significant differences. (c) Therefore, LEAK‐$J_{O_{2}}$ in the PGR group caused a significant reduction (*P* = 0.012) in the respiratory control ratio (RCR) of PGR mice compare to CON. (d) PGR showed diet × sex × age interaction in LEAK‐$J_{H_{2}O_{2}}$ emission (*P* = 0.001). At PN22 in the presence of P/M, the PGR–F had higher emission than CON–F, but at PN80 the PGR–M had elevated emission over CON–M. A diet effect (*P* = 0.008) also revealed PGR had higher $J_{H_{2}O_{2}}$ emission with P/M vs. CON. An age effect (*P* = 0.036) showed PN80 emission was higher than PN22 with P/M. (e) During P/M ADP_max_, an interaction (*P* = 0.028) was seen with $J_{H_{2}O_{2}}$ emission being elevated at PN22 in the PGR–F compared to the CON–F, but at PN80 the PGR–M had higher $J_{H_{2}O_{2}}$ emission than CON–M. Values are means ± SD. Three‐way ANOVAs with the main effects of diet (CON vs. PGR), sex (male vs. female) and age (PN22 vs. PN80) with a Tukey HSD multiple comparison test were used. Levels within the figure not connected by the same letter (A, B or C) represent statistical significance (*P* < 0.05). *P < 0.05, **P < 0.01: significantly different from CON group. †Effect of age, representing that PN80 is significantly different from PN22. PN22–CON–Male: *n* = 4; PN22–CON–Female: *n* = 4; PN22–PGR–Male: *n* = 3; PN22–PGR–Female: *n* = 5; PN80–CON–Male: *n* = 3; PN80–CON–Female: *n* = 6; PN80–PGR–Male: *n* = 4; PN80–PGR–Female: *n* = 3.

LEAK‐$J_{O_{2}}$ in the PGR group was elevated but not statistically significant (*P* = 0.068, Figure 4a) when compared to CON. During ADP‐$J_{O_{2}}$, there were no significant differences between flux of CON and PGR in the presence of P/M (Figure 4b). Therefore, LEAK‐$J_{O_{2}}$ in the PGR groups (both PN22 and PN80 together) caused a significant 11.5% reduction (*P* = 0.012) in the respiratory control ratio (RCR) (Figure 4c) of PGR when compared to CON.

PGR caused a significant interaction (diet × sex × age) effect in LEAK‐$J_{H_{2}O_{2}}$ emission (*P* = 0.001, Figure 4d), indicating at PN22, the PGR–F had more emission than CON–F, but at PN80 the PGR–M had more emission than CON–M. A diet effect (*P* = 0.008) also revealed PGR had higher $J_{H_{2}O_{2}}$ emission in the presence of P/M across both time points compared to CON. Lastly, with P/M, an age effect (*P* = 0.036) showed PN80 $J_{H_{2}O_{2}}$ emission was higher than PN22.

During P/M ADP‐$J_{H_{2}O_{2}}$, a significant interaction effect (*P* = 0.028, Figure 4e) was seen with emission being elevated at PN22 in the PGR–F compared to the CON–F, but at PN80, the PGR–M had higher $J_{H_{2}O_{2}}$ emission than CON–M. *Post hoc* analysis of this interaction revealed no significant individual comparisons.

#### 3.3.2 Succinate/rotenone

**FIGURE 5:**
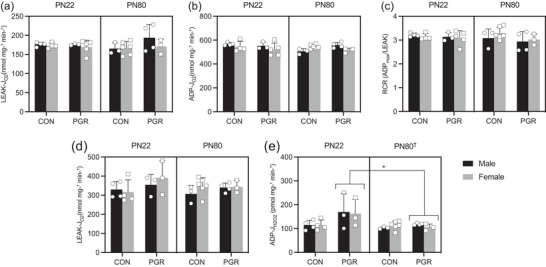
Respirometry ($J_{O_{2}}$) and fluorimetry ($J_{H_{2}O_{2}}$) with succinate/rotenone (S/R: FADH_2_ linked: complex II) in CON and PGR at PN22 and PN80. (a–d) No significant changes (a) on LEAK‐$J_{O_{2}}$, (b) ADP‐$J_{O_{2}}$, (c) RCR, or (d) LEAK‐$J_{H_{2}O_{2}}$. (e) However, during S/R ADP‐$J_{H_{2}O_{2}}$ a diet effect (*P* = 0.036) showed higher $J_{H_{2}O_{2}}$ emission in the PGR vs. CON. An age effect (*P* = 0.025) was found with higher emission at PN22 vs. PN80, in the presence of S/R. At PN22 with S/R, the PGR group showed higher emission when compared to PN22–CON, PN80–CON and PN80–PGR, but this was not significant (*P* = 0.082). Values are means ± SD. Three‐way ANOVAs with the main effects of diet (CON vs. PGR), sex (male vs. female) and age (PN22 vs. PN80) with a Tukey HSD multiple comparison test were used. *P < 0.05, significantly different from CON group. †Effect of age, representing that PN22 is significantly different from PN80. PN22–CON–Male: *n* = 4; PN22–CON–Female: *n* = 4; PN22–PGR–Male: *n* = 3; PN22–PGR–Female: *n* = 5; PN80–CON–Male: *n* = 3; PN80–CON–Female: *n* = 6; PN80–PGR–Male: *n* = 4; PN80–PGR–Female: *n* = 3.

PGR did not have a significant effect (*P* > 0.05) on $J_{O_{2}}$ when using S/R during LEAK (Figure 5a), ADP (Figure 5b) or RCR (Figure 5c) compared to CON groups. Additionally, there were no significant differences seen during LEAK‐$J_{H_{2}O_{2}}$ in the presence of S/R (Figure 5d).

During S/R ADP‐$J_{H_{2}O_{2}}$, a significant diet effect (*P* = 0.036, Figure 5e) showed higher $J_{H_{2}O_{2}}$ emission across both time points in the PGR versus CON. Additionally, in the presence of S/R, an age effect (*P* = 0.025) was found with higher emission at PN22 versus PN80. At PN22, the PGR group showed higher emission with S/R when compared to PN22–CON, PN80–CON and PN80–PGR, but this interaction was not significant (*P* = 0.082).

#### 3.3.3 Palmitoylcarnitine/malate

**FIGURE 6:**
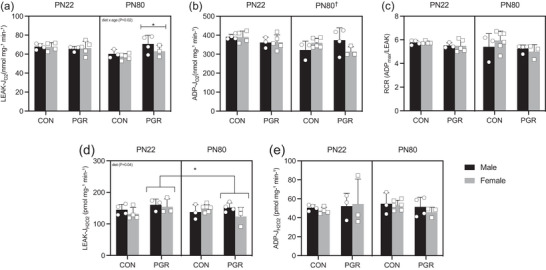
Respirometry ($J_{O_{2}}$) and fluorimetry ($J_{H_{2}O_{2}}$) with palmitoylcarnitine/malate (PC/M: fatty acid oxidation) in CON and PGR at PN22 and PN80. (a) PGR showed a diet × age interaction (*P* = 0.021) at PN80–LEAK‐$J_{O_{2}}$ compared to the CON. (b) During PC/M ADP‐$J_{O_{2}}$, PGR had a near significant interaction (*P* = 0.072) with the PN22–PGR–M having lower $J_{O_{2}}$ vs. PN22–CON–M, but at PN80 the PGR–F had lower $J_{O_{2}}$ vs. CON–F. An age effect with PC/M (*P* = 0.016) revealed PN22‐$J_{O_{2}}$ was higher than PN80. (c) PC/M RCR was not significant (*P* = 0.066). (d) PC/M LEAK‐$J_{H_{2}O_{2}}$ showed a diet effect (*P* = 0.043) with PGR having higher emission than CON. (e) There were no significant differences in ADP‐$J_{H_{2}O_{2}}$ emission between groups. Values are means ± SD. Three‐way ANOVAs with the main effects of diet (CON vs. PGR), sex (male vs. female), and age (PN22 vs. PN80) with a Tukey HSD multiple comparison test were used. Levels within the figure not connected by the same letter (A, B, or C) represent statistical significance (*P* < 0.05). *P < 0.05, significantly different from CON group. †Effect of age, representing that PN22 is significantly different from PN80. PN22–CON–Male: *n* = 4; PN22–CON–Female: *n* = 4; PN22–PGR–Male: *n* = 3; PN22–PGR–Female: *n* = 5; PN80–CON–Male: *n* = 3; PN80–CON–Female: *n* = 6; PN80–PGR–Male: *n* = 4; PN80–PGR–Female: *n* = 3.

PGR caused a diet × age interaction effect (*P* = 0.021, Figure 6a) leading to a 13.8% increased LEAK‐$J_{O_{2}}$ compared to the CON at PN80. No significant differences were seen during PN22‐LEAK‐$J_{O_{2}}$.

During PC/M ADP‐$J_{O_{2}}$, PGR caused an interaction effect (*P* = 0.072, Figure 6b) for the three‐way diet × sex × age with the PN22–PGR–M having lower $J_{O_{2}}$ versus PN22–CON–M, but at PN80 the PGR–F had lower $J_{O_{2}}$ versus CON–F. With PC/M, a significant age effect (*P* = 0.016) also showed that PN22‐$J_{O_{2}}$ was higher than PN80, but this finding was not statistically significant. The PC/M RCR showed PGR had a 6.5% reduction (5.3 ± 0.4) when compared to the CON (5.7 ± 0.6), but this finding was not significant (*P* = 0.066, Figure 6c).

During LEAK‐$J_{H_{2}O_{2}}$ (Figure 6d), PGR caused a significant diet effect (*P* = 0.043), having higher emission than CON, across both time points in the presence of PC/M. During ADP‐$J_{H_{2}O_{2}}$ in the presence of PC/M (Figure 6e), there were no significant differences (*P* > 0.05) in $J_{H_{2}O_{2}}$ emission between groups.

### 3.4 Western blotting for mitochondrial proteins related to observed bioenergetic abnormalities

To support the respirometry and fluorimetry results, we probed for proteins related to heightened LEAK and ROS emission at PN22 and PN80 (UCP3, PRDX6, VDAC and ANT1).

#### 3.4.1 UCP3 protein abundance

**FIGURE 7:**
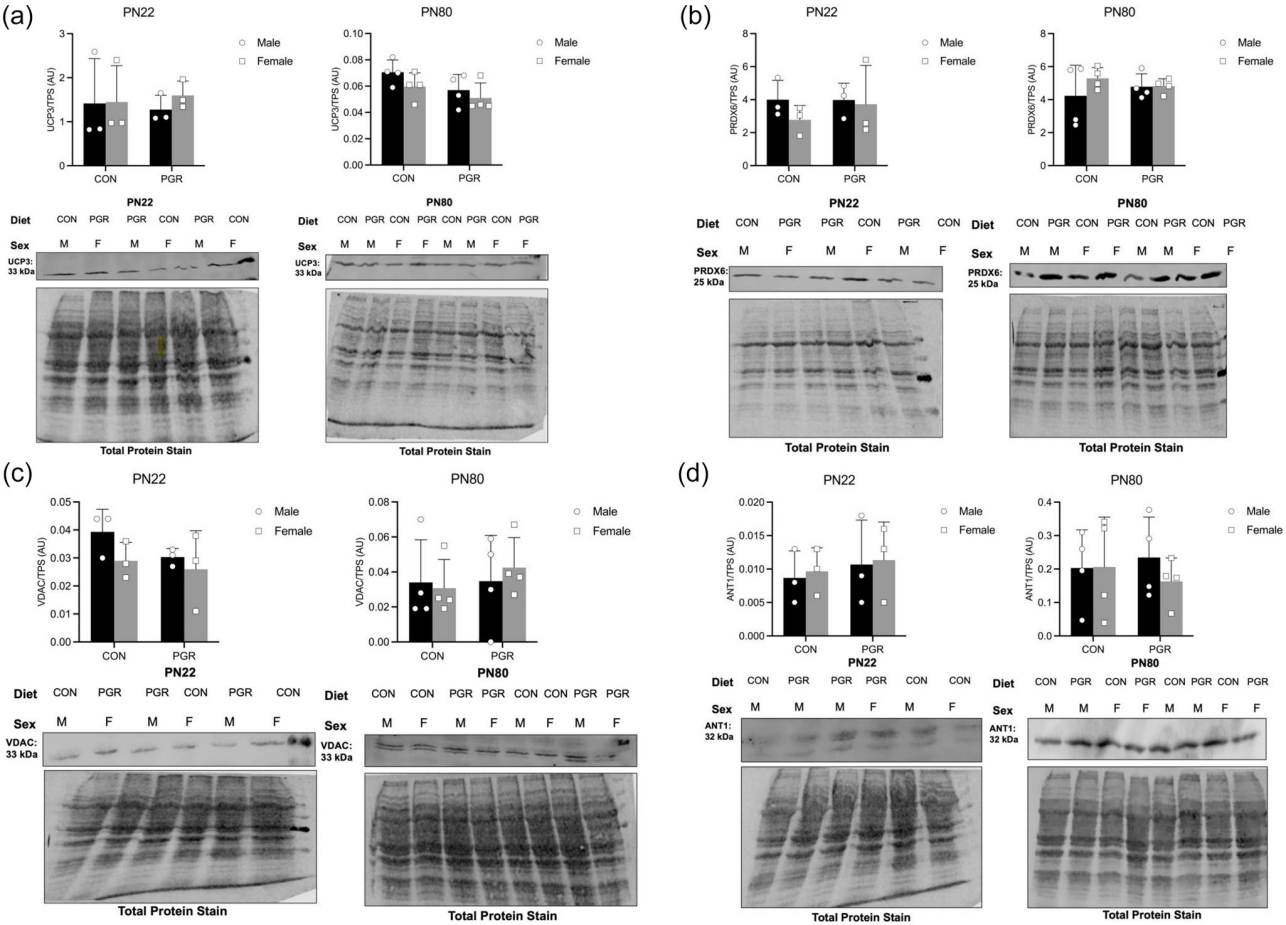
Relative mitochondrial protein abundance during development (PN22) and adulthood (PN80) of UCP3, PRDX6, VDAC and ANT1 in CON and PGR groups. (a–d) At PN22 and PN80, no significant differences were seen in the protein abundance of UCP3, PRDX6, VDAC or ANT1. Values are means ± SD. PN22–CON–Male: *n* = 3; PN22–CON–Female: *n* = 3; PN22–PGR–Male: *n* = 3; PN22–PGR–Female: *n* = 3; PN80–CON–Male: *n* = 4; PN80–CON–Female: *n* = 4; PN80–PGR–Male: *n* = 4; PN80–PGR–Female: *n* = 4.

At PN22, there were no significant alterations to UCP3 protein abundance (*P* > 0.05). At PN80, there was a non‐significant reduction (*P* = 0.061) in the relative protein abundance of UCP3 between PGR and CON (Figure 7a). The PN80 UCP3 comparison of sex and interaction of diet × sex was also non‐significant (*P* > 0.05).

#### 3.4.2 PRDX6 protein abundance

Despite previous literature reporting reduced PRDX6 levels in PGR mice, our data revealed no significant diet, sex or interactions (*P* > 0.05) in PRDX6 protein abundance at PN22 or PN80 (Figure 7b).

#### 3.4.3 VDAC protein abundance

PGR showed no significant alterations (*P* > 0.05) to VDAC protein abundance (Figure 7c) at PN22 or PN80.

#### 3.4.4 ANT1 protein abundance

At PN22 and PN80, PGR showed no significant changes (*P* > 0.05) to the relative protein abundance of ANT1 (Figure 7d).

## 4 DISCUSSION

Nearly 161 million children annually experience growth restriction through a limiting nutritive environment in utero or in postnatal life (Barker, 1997, 2000, 2007; Barker, Osmond, et al., 1989). Growth restriction during postnatal development can increase the onset of chronic disease in adulthood, compared to the non‐growth‐restricted population (Leszczynski et al., 2019; Visker et al., 2020). Specifically, the DOHaD literature frequently demonstrates that growth restriction in early life causes cardiovascular impairments increasing disease pathology in adult humans, non‐human primates, farm animals and rodents (Barker, 2000; Barker, Winter, et al., 1989; Osmond et al., 1993; Thornburg, 2015).

In support of previous literature, we demonstrated that PGR body mass in adulthood (PN80 in this investigation) is markedly reduced compared to unrestricted controls (Figure 3a,b) (Ferguson et al., 2019; Leszczynski et al., 2019; Pendergrast et al., 2020; Visker et al., 2020, 2018). Absolute, but not relative, heart mass of PGR was less than CON at both PN22 and PN80, indicating hearts were proportionate for the size of the animals (Figure 3c,d). Interestingly, although the PGR hearts are proportionate for their body size, our data also show that in the heart the mitochondrial content of the PGR mice is reduced by 26.3%, which may contribute to cardiac pathology (reduced LV mass, reduced cardiac output and increased isovolumetric contraction/relaxation time) as evident from studies using identical mice and experimental design (Ferguson et al., 2019; Pendergrast et al., 2020; Visker et al., 2020, 2018). Additionally, in support of previous DOHaD research, tibia lengths of PGR mice were reduced at PN22 and PN80, which typically associates with less overall growth and is suggestive of impaired lean tissue growth (Fiorotto et al., 2014).

The publications surrounding metabolism and growth restriction show intrauterine growth restriction alters redox ratios, increases reliance on glycolysis and reduces lipid oxidation in adult offspring (Beauchamp et al., 2015; Dimasi et al., 2021; Vaughan et al., 2022; Wang et al., 2013). Presently, there are no investigations exploring PGR and myocardial energetics in adulthood. However, Beauchamp et al. (2015) showed the adverse effect of a 50% caloric reduction during gestation on adult female offspring with impairments to myocardial energy metabolism, and demonstrated that this may have long‐term consequences for CV function and disease in adulthood. Their study reported reductions in fatty acid oxidation in the presence of octanoylcarnitine in adult females but did not obtain respirometry or fluorimetry data with different substrates or during early development. Since PGR is related to myocardial impairment potentially originating from impaired cardiac metabolism, we designed our experiments to analyse OxPhos and myocardial mitochondrial energetics of PGR mice (males and females) of different ages (PN22 and PN80). Additionally, we modelled various scenarios in vitro related to cardiac substrate utilization (P/M, S/R and PC/M) and analysed key proteins involved in OxPhos such as UCP3, PRDX6, VDAC and ANT1.

### 4.1 Respirometry ($J_{O_{2}}$) and fluorimetry ($J_{H_{2}O_{2}}$)

LEAK‐$J_{O_{2}}$ represents the mitochondria respiring to maintain resting membrane potential, and when elevated, may indicate a reduced proton motive force (*∆P*) or altered mitochondrial membrane potential (*∆*Ψ) (Brand & Nicholls, 2011). Based upon our results, the PGR group had an 11.5% and 13.8% higher LEAK‐$J_{O_{2}}$ compared to controls when their myocardial mitochondria were fuelled with P/M (Figure 4) and PC/M (Figure 6), respectively. Although there is no clinically established threshold, reduced ∆*P* through elevated LEAK‐$J_{O_{2}}$ is related to cardiac disease pathology (Divakaruni & Brand, 2011). Quarrie et al. (2011) reported a two‐fold increase during LEAK‐$J_{O_{2}}$ in isolated rat mitochondria with ischaemia–reperfusion injury compared to non‐ischaemic controls. The PGR group in this study did not show LEAK‐$J_{O_{2}}$ as high as reported by Quarrie et al., but the higher proton leak, in the presence of P/M, did lead to a significant 11.54% reduction in RCR (Figure 4c).

RCR is a useful measure of the dynamic range of OxPhos. Without ADP, mitochondria idle at low LEAK states but rapidly respond to ADP and produce ATP (Brand & Nicholls, 2011). Sharov et al. (2000) previously showed in humans with ischaemic cardiomyopathy a 25–30% reduction in RCR, compared with normal donor hearts. Lemieux et al. (2011) specified LEAK respiration increases in response to stress, thus decreasing RCR, and showed a ∼12% complex I‐ and complex II‐linked reduction to RCR in human heart disease patients with no or mild heart failure undergoing heart surgery (average age: 64 years). Therefore, our data closely align with the general observations shown in Lemieux et al. namely, the PGR myocardial mitochondria in our study had a higher permeability to protons, and hence the heightened LEAK‐$J_{O_{2}}$ lowered the OxPhos dynamic range (↓ RCR with P/M), which has been associated with modest CVD such as reduced cardiac output and diastolic dysfunction (Cheng et al., 2017).

It has previously been shown using mass spectrometry that PGR mouse hearts have reduced proteomic abundance of PRDX6 at PN21, which may alter the cardiomyocytes’ ability to handle ROS emission (Visker et al., 2020). Although we did not observe the changes previously reported in PRDX6 levels, our results suggest an inability for the PGR hearts to mitigate ROS emission, potentially from a different mechanism. For example, the $J_{H_{2}O_{2}}$ was significantly elevated when PGR myocardial mitochondria were fuelled with carbohydrate‐linked metabolites (P/M; Figure 4d), TCA intermediates (S/R; Figure 5e) and fatty acids (PC/M; Figure 6d), which may represent a compensatory mechanism with over‐activation of pyruvate dehydrogenase. It is widely accepted that mitochondrial ROS emission can lead to oxidative damage (Holmberg et al., 1991), and even minor chronic elevations of ROS emission throughout the lifespan can lead to mitochondrial dysfunction, chronic oxidative stress and the pathogenesis of CVD (Brand & Nicholls, 2011).

Elevated oxidative stress has been shown to disrupt intracellular calcium handling (Holmberg et al., 1991), which induces cardiomyocyte electrophysiological alterations and may precipitate ventricular arrhythmias (Cerbai et al., 1991). This disease pathology closely parallels cardiac impairments (dysregulated calcium flux and prolonged QRS complexes) seen in PGR mouse hearts in adulthood (Ferguson et al., 2019; Visker et al., 2018). The significance of LEAK‐$J_{H_{2}O_{2}}$ in the murine heart has been a topic of debate since the heart is almost always active. However, our findings show that there is a higher mitochondrial $J_{H_{2}O_{2}}$ at LEAK (P/M and PC/M) and when stimulated with ADP (S/R), which indicates at least some level of chronic oxidative stress that may play a role in the development of CVD associated with PGR (Cheng et al., 2017).

### 4.2 Abundance of mitochondrial proteins related to higher LEAK respirometry

Based upon results from respirometry and fluorimetry, which indicated impairments to LEAK state respirometry and heightened ROS emission, we probed for mitochondrial proteins such as UCP3 (proton gradient), PRDX6 (antioxidant), VDAC (metabolite transport and apoptosis) and ANT1 (transports ADP and ATP across the inner mitochondrial membrane) at PN22 and again at PN80.

Interestingly, our results did not reveal any differences in the relative protein abundance of these selected targets between PGR and CON groups. Although there were no diet effects seen in protein abundance, it is possible that post‐translational modifications have occurred that could affect function, localization and stability of these targets, but more research is needed.

These results suggest that PGR and CON hearts have the same abundance of UCP3, PRDX6, VDAC and ANT1 but protein function is potentially compromised leading to mitochondrial impairments seen from respirometry and fluorimetry. Another explanation is that additional mechanisms within OxPhos might lead to respiratory deficits in the PGR mitochondria such as allosteric regulation, post‐translational modifications and availability of substrates or co‐factors.

### 4.3 Limitations

The results from this investigation reflect the mitochondrial physiology from whole hearts, and it is possible that there may be differences in the right or left side of the heart. Mitochondria were incubated at saturating O_2_ concentration, which is much higher than the O_2_ concentration experienced by mitochondria in tissues. Even so, the elevated ROS emission rates observed from the PGR mitochondria will be maintained at lower, more physiologically relevant O_2_ concentrations since the production of ROS is linearly proportional to the O_2_ concentration (Duong et al., 2020, 2021). As a result, we expect our results to be confirmed by oxidative stress markers such as lipid peroxidation, protein carbonylation and/or protein sulfenation.

Although we used a well‐established protocol (Bazil et al., 2016; Duong et al., 2020, 2021; Vinnakota et al., 2016), which has been proven to reproducibly yield high‐quality mitochondrial isolates, we did not measure cytochrome *c* to assess mitochondrial integrity in this study.

### 4.4 Conclusion

PGR alters myocardial mitochondrial energetics at PN22 and again at PN80 in several different ways. Based upon these results, impairments in the PGR mitochondria are mostly complex I and fatty acid oxidation dependent when fuelled with P/M or PC/M. Reduced mitochondrial efficiency (↓ RCR: P/M, ↓11.54%; and PC/M, ↓6.5%) was indicated during LEAK‐$J_{O_{2}}$. These deficits indicate metabolic inflexibility and may reduce proton motive force, and future research should measure mitochondrial membrane potential in PGR myocardial mitochondria. Additionally, PGR mitochondria have significantly elevated $J_{H_{2}O_{2}}$ at PN22 and PN80, indicating a low but chronic oxidative stressed environment. Oxidative stress from an increase in mitochondrial ROS emission is a part of many CVD mechanisms (Tain & Hsu, 2017). Collectively, these results indicate impairments to mitochondrial function and myocardial energetics in PGR mouse hearts.

## AUTHOR CONTRIBUTIONS

Joseph R. Visker, Jason N. Bazil and David P. Ferguson designed and developed the overall research plan. Joseph R. Visker, Eric C. Leszczynski, Ashley C. McPeek, Melissa A. Quinn, Seong Hyun Kim and Austin G.Wellette–Hunsucker conducted hands‐on experiments for data collection. David P. Ferguson and Jason N. Bazil provided essential reagents, and materials. Joseph R. Visker, Eric C. Leszczynski, Ashley C. McPeek, Melissa A. Quinn, Austin G.Wellette–Hunsucker, Seong Hyun Kim., Jason N. Bazil and David P. Ferguson analysed data and performed statistical analyses. Joseph R. Visker, Eric C. Leszczynski, Austin G.Wellette–Hunsucker, Jason N. Bazil and David P. Ferguson wrote the manuscript. All authors have read and approved the final version of this manuscript and agree to be accountable for all aspects of the work in ensuring that questions related to the accuracy or integrity of any part of the work are appropriately investigated and resolved. All persons designated as authors qualify for authorship, and all those who qualify for authorship are listed.

## CONFLICT OF INTEREST

The authors declare no conflicts of interest. The results of this study are presented clearly, honestly and without fabrication, falsification or inappropriate data manipulation, and results of the present study do not constitute endorsement by the publisher.

## Data Availability

The datasets during and/or analysed during the current study are available from the corresponding author on reasonable request.
